# Xanthogranulomatous Pyelonephritis Causing Renocolic Fistula Presenting as Symptomatic Anemia

**DOI:** 10.7759/cureus.4947

**Published:** 2019-06-19

**Authors:** Laith Numan, Harris Zamir, Nedaa M Husainat, Mohammad Tahboub

**Affiliations:** 1 Internal Medicine, University of Missouri-Kansas City School of Medicine, Kansas City, USA; 2 Kidney Institute, University of Kansas Hospital & Medical Center, Kansas City, USA; 3 Internal Medicine, University of Missouri-Kansas City l Saint Luke's Health System, Kansas City, USA

**Keywords:** renocolic fistula, xanthogranulomatous pyelonephritis (xgp), anemia, kidney stones, colonoscopy findings

## Abstract

Renocolic fistula is a rare clinical finding that is most commonly iatrogenic after surgical intervention. Herein, we present a case of renocolic fistula secondary to xanthogranulomatous pyelonephritis (XGP) with a subtle presentation as anemia. A 40-year-old female was found to have a hemoglobin of 6.5 g/dL after presenting for worsening fatigue. A urinalysis was remarkable for numerous white blood cell (WBC), positive bacteria, and nitrite. As part of her anemia workup, an esophagogastroduodenoscopy (EGD) was done which was normal while a colonoscopy showed a fistula opening with surrounding nodularity close to the splenic flexure of the colon. A computed tomography (CT) scan of the abdomen and pelvis with contrast showed chronic left kidney pyelonephritis with multiple contiguous abscesses in the inferior left kidney in addition to a staghorn calculus concerning for XGP. The patient was started on antibiotics and underwent laparotomy with repair of the renocolic fistula, partial omentectomy, and left nephrectomy. She tolerated the surgery well and was discharged with a stable hemoglobin. XGP is a rare type of chronic pyelonephritis that is usually a result of chronic obstruction by an infected stone. Spontaneous renocolic fistulas are rare nowadays with the advancement in antibiotics and renal stones treatment.

## Introduction

Xanthogranulomatous pyelonephritis (XGP) is an uncommon cause of chronic pyelonephritis arising from obstruction of the kidney by infected renal stones [1]. Granulomatous tissue invades the kidney, and resultant lipid-laden macrophages destroy the renal tissue [2]. These patients commonly present with flank pain, and a unilateral renal mass felt on the physical exam [3]. A rare sequela of this uncommon disease is the renocolic fistula [2]. A renocolic fistula is usually associated with primary bowel pathologies, including inflammatory bowel diseases and malignancies [4]. That is why, without any suspicion for a primary bowel pathology, a diagnosis of renocolic fistula is rarely pursued.

We report a case of renocolic fistula caused by XGP in a patient that presented without a flank pain. Instead, our patient presented with symptomatic anemia and dysuria. XGP was on the differential after a colonoscopy was performed and revealed a renocolic fistula.

## Case presentation

A 40-year-old female with no significant past medical history who was seen by her primary care physician for fatigue and workup revealed low hemoglobin, so she was asked to go to the emergency department (ED) for further evaluation. The patient has been complaining of fatigue, nausea, diarrhea, dysuria, and urinary frequency for more than seven days prior to presentation. On physical examination, the patient was pale, had mild abdominal tenderness, and left flank fullness was felt. Labs were repeated in the ED and were remarkable for a hemoglobin of 6.5 g/dL (down from 10 g/dL three months prior), white blood cell (WBC) count of 18 x 10^9^/L, creatinine of 1.87 mg/dL (baseline is 1 mg/dL) with blood urea nitrogen (BUN) of 24 mg/dL. Urinalysis was remarkable for numerous WBC, positive bacteria, and nitrite. The patient received one unit of packed red blood cells and was started on ceftriaxone for urinary tract infection (UTI). No source of active bleeding was identified.

**Figure 1:**
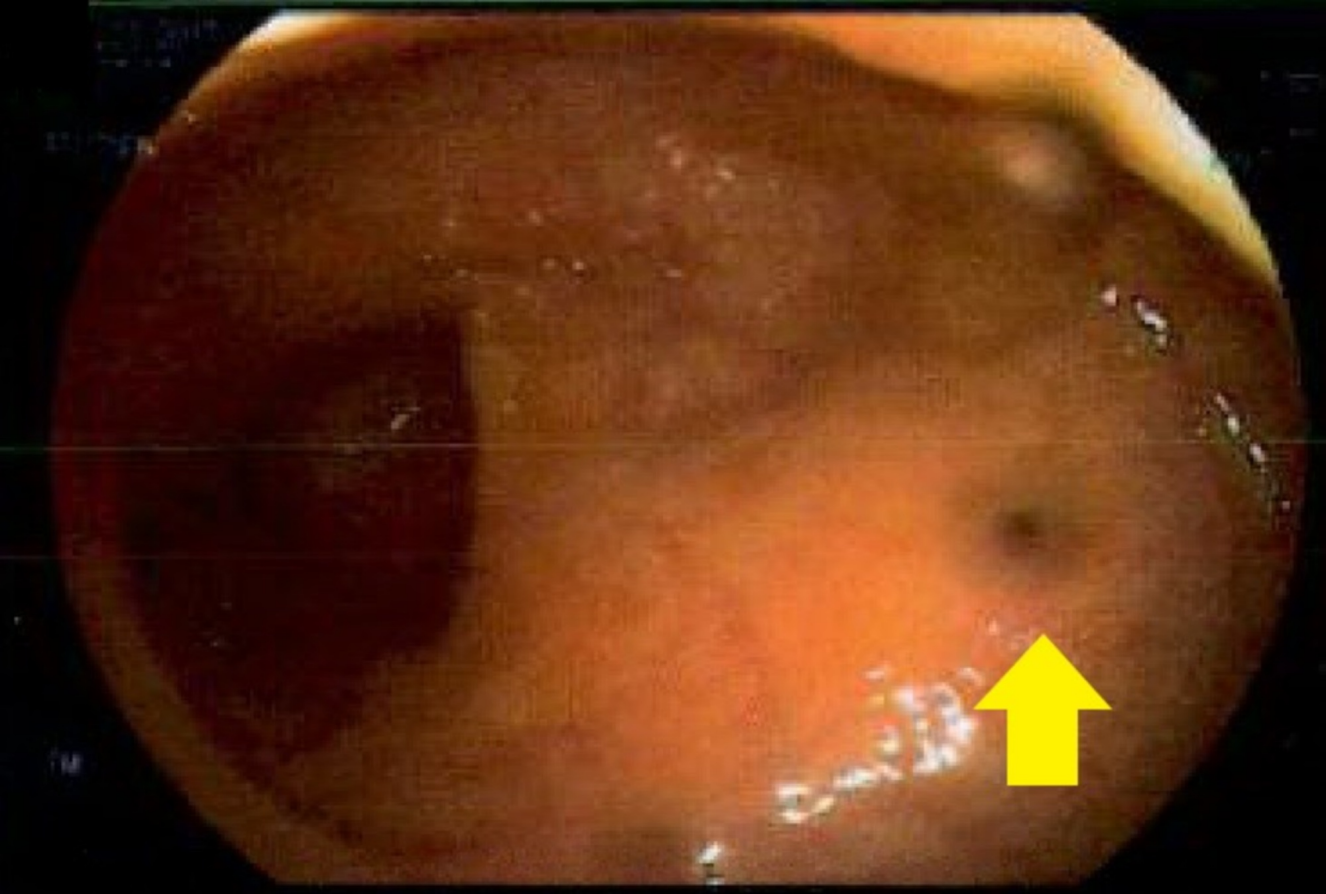
Colonoscopy image showing the renocolic fistula opening with surrounding nodularity

Gastroenterology team was consulted for the anemia, and they performed esophagogastroduodenoscopy (EGD) which was normal. Subsequently, they performed a colonoscopy which detected a fistula opening with surrounding nodularity close to the splenic flexure of the colon with a small amount of pus noted coming out of it (Figure 1).

**Figure 2:**
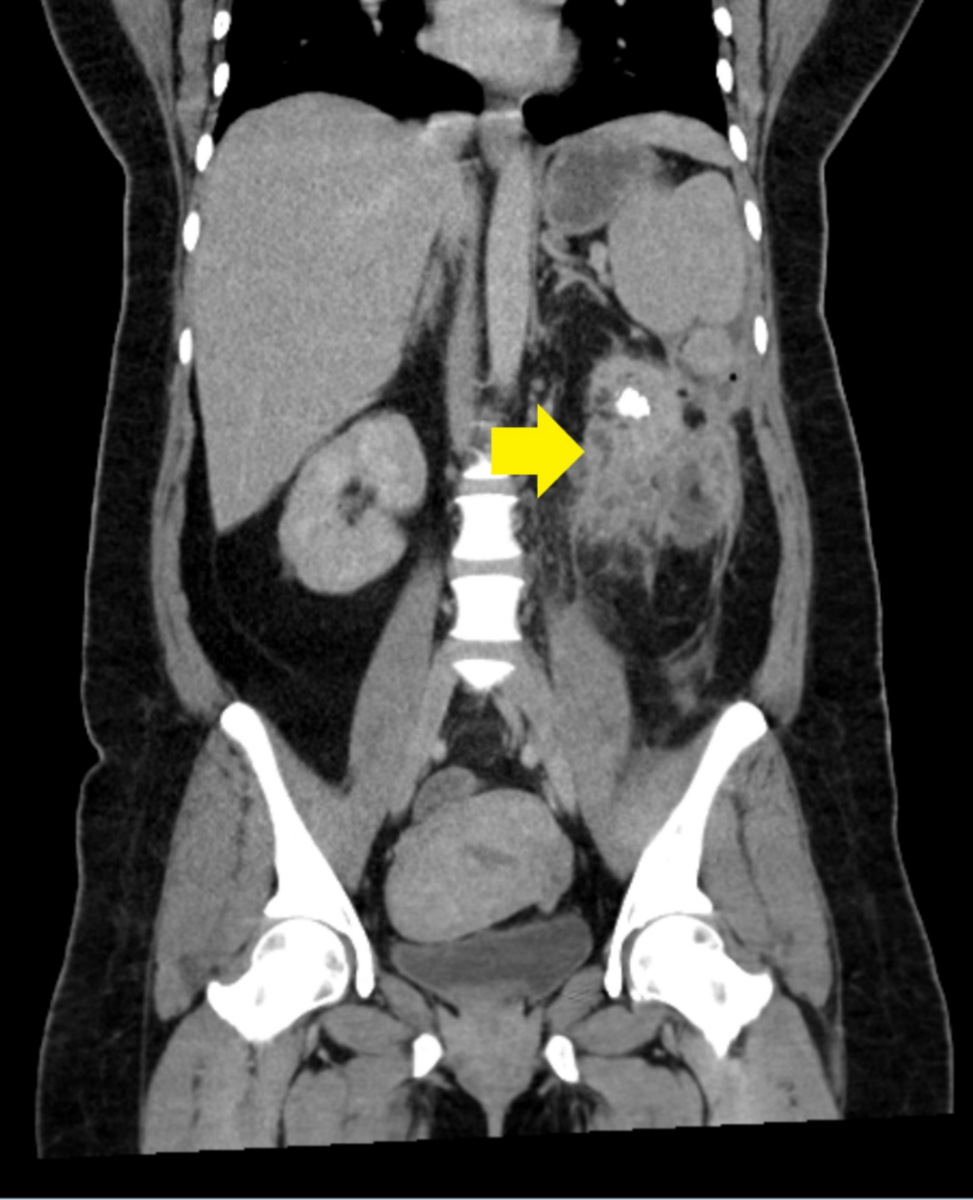
Computed tomography scan of the abdomen and pelvis showing xanthogranulomatous pyelonephritis

**Figure 3:**
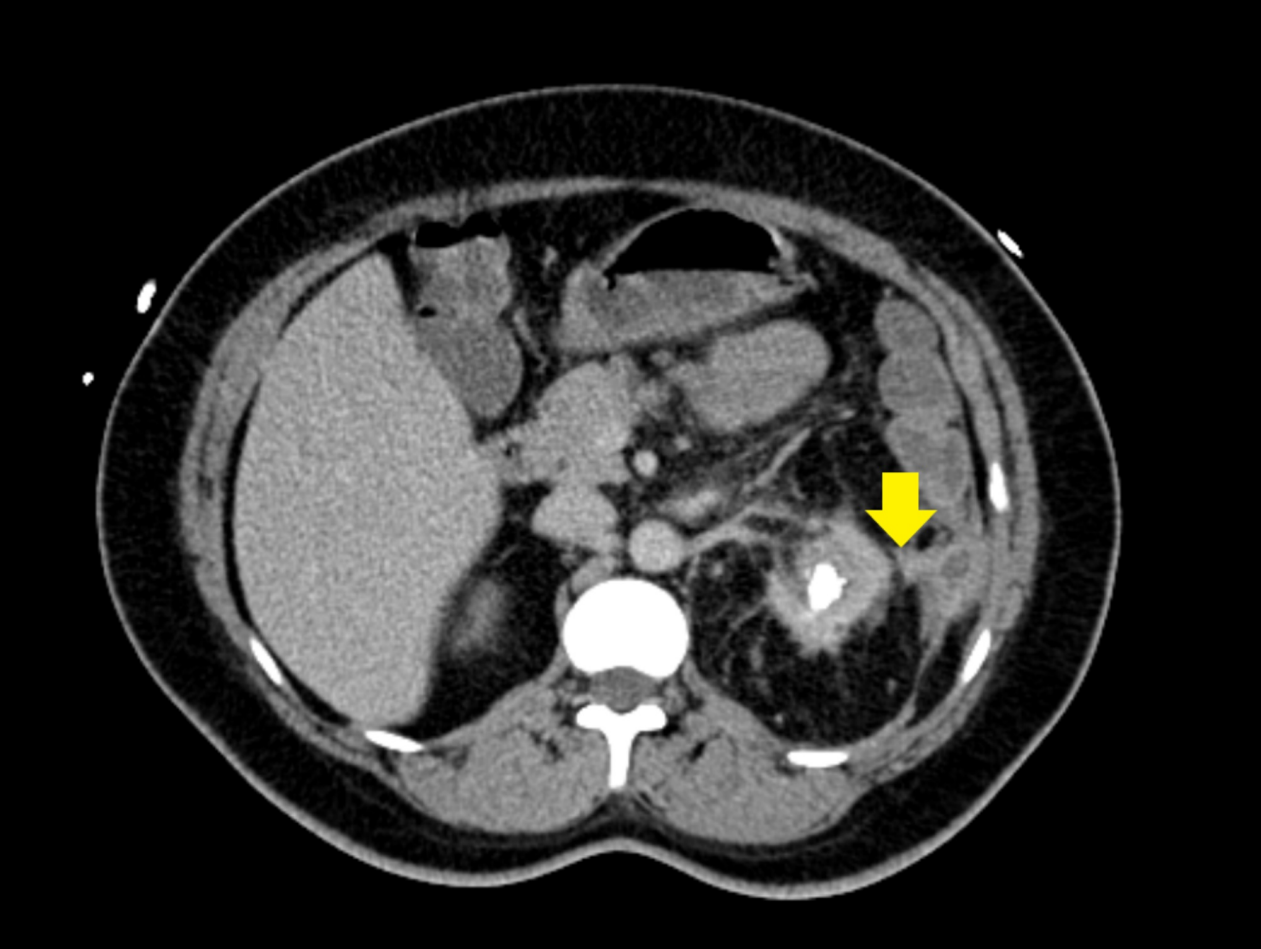
Computed tomography scan of the abdomen and pelvis showing the renocolic fistula

Shortly after, the patient spiked a fever of 102.7F. C-reactive protein (CRP) and erythrocyte sedimentation rate (ESR) were both elevated. Based on the colonoscopy findings and the new fever, a computed tomography (CT) scan of the abdomen and pelvis with contrast was performed and it revealed chronic left kidney pyelonephritis with multiple contiguous abscesses in the inferior left kidney with a staghorn calculus concerning for XGP; it also showed the fistula between the left kidney and the splenic flexure of the colon (Figures 2-3). The patient underwent exploratory laparotomy, takedown and repair of renocolic fistula, partial omentectomy, and left nephrectomy by urology and general surgery teams. The patient had an uncomplicated postoperative course and was discharged home on postoperative day six with stable hemoglobin of 8 g/dL.

## Discussion

XGP is a rare cause of chronic pyelonephritis caused by obstruction of the kidney by infected renal stones [1]. The actual incidence of XGP is estimated to be around 1% of renal infections cases, although there is no valid statistics to validate this number [1]. The body responds with inflammation in the affected kidney in response to this infection, which is carried out in the form of lipid-laden macrophages and accompanying granulomatous tissue. The foamy histiocytes destroy the kidney and which leads to chronic pyelonephritis [2].

This rare disease is most prevalent in middle-aged women who tend to be at higher risk for recurrent UTIs [4]. The presentation is usually similar to pyelonephritis, including flank pain, fever, and a unilateral renal mass palpated on physical examination [3]. Laboratory testing is typically nonspecific and occasionally reveals anemia and elevation in inflammatory markers [5]. Urinalysis is routinely suggestive of a UTI, and the most common bacterias isolated in urine culture are Escherichia coli, Proteus mirabilis, Pseudomonas species, Enterococcus faecalis, and Klebsiella [5].

In the case we reported, the patient presented with symptomatic anemia and dysuria and had none of the other common signs or symptoms of XGP. In the period of initial management, there was no indication for a CT scan. However, a CT scan and subsequent pathology confirmed the diagnosis of XGP. CT scan usually demonstrates round areas of low density surrounded by a rim of contrast medium [6]. This finding often referred to as the "bear's paw sign" [7]. This classic presentation was seen in our patient.

The lesion is then routinely staged based on the extension of the disease. Stage 1: localized to the renal parenchyma; Stage 2: involves the perinephric fat; Stage 3: involves the perinephric fat and/or abdominal wall, and Advanced stage: involves the adjacent gastrointestinal tract [1]. Our patient was easily staged as advanced disease as he had renocolic fistula. In XGP, growing inflammation and increased pressure inside the kidney results in necrosis of the thinning cortex and formation of an abscess. This abscess continues to grow, perforating neighboring structures. In most cases, renocolic fistulas are seen, however, renocutaneous and renobronchial fistulas have also been described before [8].

Staghorn calculi are classically the renal stones infected in XGP and were seen in our patient as well [9]. This first event drives the pathology of XGP and explains the foamy histiocytes seen on histopathology to confirm the diagnosis. The resultant inflammatory response to the infected stones with lipid-laden macrophages results in yellow necrotic tissue seen in infected kidneys [6]. It is proposed that a dysfunction in the macrophages ability to process bacteria results in the destructive inflammation, but the intricacies of the pathology are not well understood [10].

Due to the similarities in clinical presentation and plain film findings, XGP can be initially misdiagnosed as renal carcinoma. However, a history of UTIs and CT findings easily differentiate these diagnoses [11]. In this regard, XGP is a differential that physicians should regard highly in a patient presenting with these typical symptoms. This is additionally important because the prognosis of XGP relies on early diagnosis and management of the disease.

Initial management of XGP usually involves initial antibiotic therapy for the treatment of the acute infection. However, the definitive treatment is surgical with nephrectomy removing all the involved tissues and any extensions of the disease, including fistulas [12]. Although Hippocrates is credited with first describing a renal abscess invading the intestinal tract, the first reported renocolic fistula was in 1841 by Rayer. Since then, the advent of antimicrobial therapy and CT scan have made the pathology rare [13].

This case was presented as a poster at the American College of Gastroenterology annual meeting (Poster: Tahboub L, Abughanimeh O, Numan L, et al. Xanthogranulomatous Pyelonephritis Causing a Renocolic Fistula Presenting as Symptomatic Anemia. American College of Gastroenterology 2018 Annual Scientific Meeting Abstracts. Philadelphia, Pennsylvania; 2018).

## Conclusions

XGP is a rare type of chronic pyelonephritis that is usually a result of chronic obstruction by an infected stone. Spontaneous renocolic fistulas are rare, especially with the advancement in antibiotics and renal stones treatment. XGP can present with mild anemia and dysuria. However, imaging is necessary for diagnosis.
